# Timing of Artificial Insemination Using Sexed or Conventional Semen Based on Automated Activity Monitoring of Estrus in Holstein Heifers

**DOI:** 10.3390/ani13192994

**Published:** 2023-09-22

**Authors:** Christie Marie Tippenhauer, Jan-Lukas Plenio, Augusto Madureira, Wolfgang Heuwieser, Stefan Borchardt

**Affiliations:** 1 Farm Animal Clinic, Division for Ruminants and Camelids, Unit for Reproduction Medicine and Udder Health, School of Veterinary Medicine, Freie Universitaet Berlin, 14163 Berlin, Germany; 2 Institute for Veterinary Epidemiology and Biostatistics, Freie Universitaet Berlin, 14163 Berlin, Germany; 3 Ridgetown Campus, University of Guelph, Ridgetown, ON N0P 2C0, Canada

**Keywords:** insemination time, activity monitor, pregnancy, Holstein heifer

## Abstract

**Simple Summary:**

Automated activity monitors (AAM) represent a useful tool in estrus detection to improve reproductive performance in dairy herds. There are only a few studies, however, investigating the association between the characteristics of an estrus event (i.e., the onset, peak, and end of estrus) determined by an AAM and the timing of artificial insemination (AI) in heifers, depending on the semen type used. Therefore, this observational study was conducted to determine the association between the interval from different characteristics of estrus and the timing of AI on pregnancy per AI (P/AI). Holstein heifers were fitted with a neck-mounted AAM and inseminated either with frozen conventional or sexed semen. Heifers inseminated from 9 to 32 h after the onset of estrus had the greatest P/AI, irrespective of semen type.

**Abstract:**

Investigations on the optimum timing of artificial insemination (AI) following automated activity monitoring (AAM) depending on different types of semen in heifers are limited and in part show controversial results. Therefore, the objective of this observational study was to determine the association between the timing of AI using different characteristics of estrus (i.e., the onset, peak, and end of estrus) and pregnancy per AI (P/AI) in Holstein heifers. Heifers were fitted with a neck-mounted AAM system and inseminated with frozen conventional and sexed semen. The pregnancy per AI (*n* = 4159) from 2858 heifers from six commercial dairy farms in Germany inseminated upon the alert of an AAM system was evaluated. Estrous intensity was classified based on peak activity into low (35 to 89 index value) and high (90 to 100 index value). We detected a quadratic association between the interval from the onset of estrus to AI and P/AI (*p* = 0.02). The greatest P/AI was observed for heifers inseminated from 9 to 32 h after the onset of estrus. The intervals from the peak of activity to AI and the end of estrus to AI were not associated with P/AI (*p* ≥ 0.05). Heifers inseminated with frozen conventional semen (50.1%) had a greater P/AI compared with heifers inseminated with frozen sexed semen (43.3%; *p* = 0.03). There were no interactions between the intervals from the onset, peak, or end of estrus to AI or the type of semen and the P/AI (*p* ≥ 0.05). The pregnancy per AI was not associated with estrous intensity (50.5% for low intensity vs. 53.0% for high intensity; *p* = 0.37). In conclusion, inseminating heifers between 9 and 32 h after the onset of estrus, as detected by the AAM, optimized the P/AI regardless of semen type.

## 1. Introduction

The timing of artificial insemination (AI) after the accurate detection of estrus is of major importance for high pregnancy rates. Considering biological factors such as the lifespan of sperm and oocyte, one of the major challenges is to identify the time of ovulation to achieve satisfactory pregnancy outcomes [1,2]. Using automated activity monitors (AAM), the mean interval from the onset of estrus to ovulation was approximately 29 h in Holstein heifers [3,4]. Therefore, the implementation of AAM systems in reproductive management may represent an opportunity to improve reproductive performance in dairy herds.

In a recent study from our group [5], inseminating dairy cows from 7 to 24 h after the onset of estrus yielded the greatest pregnancy per AI (P/AI), irrespective of type of semen (i.e., fresh or conventional frozen semen). Lactating cows, however, differ in their ovarian physiology due to an altered steroid metabolism [6]. In addition, when fitted with an AAM, heifers had a longer duration of increased activity compared with cows [7]. Therefore, the optimum timing of AI may differ in heifers compared with lactating cows.

Only a few studies have explored the association between the estrus characteristics determined by AAM systems and the timing of AI in heifers [3,4,8]. Macmillan et al. [4] reported a reduced probability of pregnancy for each hour increase in the interval from the onset of estrus (−3.8%) or peak activity (−4.2%) to AI when inseminating pre-synchronized Holstein heifers with conventional frozen semen. Sales et al. [8], on the other hand, noted no difference in the P/AI for Jersey heifers receiving timed AI (TAI) with conventional semen at 6.5 h versus 12.5 h before presumptive ovulation. In the latter study, however, Jersey heifers had a greater P/AI when inseminated at 6.5 h versus 12.5 h before presumptive ovulation when inseminated with sexed semen. Their finding to optimize reproductive performance by delaying AI with sexed semen (i.e., closer to the presumed time of ovulation) was similar to those of others [3,9]. Delaying AI by approximately 12 h, however, had no effect on P/AI in heifers from another study [10]. They suspected that choosing too large intervals from the onset of estrus to AI limited their ability to properly test whether delaying AI by 12 h would affect the P/AI.

Therefore, the primary objective of this observational study was to ascertain the relationship between the timing of AI and the P/AI in Holstein heifers inseminated with either sexed or conventional semen based on estrous activity markers generated by the AAM system. The optimum timing was assessed for different characteristics of an estrus event (i.e., the onset of estrus, peak activity, and end of estrus) using a neck-mounted AAM system. Furthermore, we wanted to assess the association between estrous intensity (i.e., the peak of activity) and the P/AI of heifers. We hypothesized that delaying AI after the onset of estrus and therefore inseminating closer to the end of an estrus event would improve the P/AI in heifers inseminated with sexed semen compared with conventional semen. Furthermore, we expected high estrous intensity to be associated with an increased P/AI.

## 2. Materials and Methods

### 2.1. Study Design 

This study was an observational cohort study including 5543 AIs from 3546 Holstein heifers from 1 heifer raising farm (farm 1) and 5 commercial dairy farms (farms 2–6) in northeast Germany. The study was conducted from July 2018 to October 2021. Inclusion criteria for farms were a herd size above 400 cows and/or heifers, the use of a neck-mounted AAM system (Heatime; SCR Engineers Ltd., Netanya, Israel), and a calving date available for at least one year after the date of the corresponding AI for heifers from farm 1. Herd size ranged from approximately 400 to 700 cows, with numerous replacement heifers ranging from approximately 400 to 900 per farm (Table 1). All heifers were housed in free-stall barns. All experimental procedures were approved by the Institutional Animal Care and Use Committee of the Freie Universität Berlin.

### 2.2. Automated Activity Monitoring System

On each farm, all heifers were fitted with a neck-mounted AAM system at the age of approximately 13 months. The AAM system was removed at 42 ± 3 d after AI if a heifer had no activity alert in the meantime (farm 1), stayed attached to the heifers until a confirmed pregnancy diagnosis (farms 2–4, 6), or was removed if a heifer did not get pregnant after 3 subsequent AI (farm 5). The activity data of each heifer were recorded in real time for 2 h periods by a wireless receiver box and transmitted to the accelerometer software (DataFlow II v.21.1.3.0, SCR Engineers Ltd., Rahway, NJ, USA), which was installed on the farm computer. The processing of the data was conducted as described in Tippenhauer et al. [5,11]. Raw activity data of each heifer were converted into an activity change index by calculating the difference between today’s last 2 h of raw activity and the mean of last week’s activity in the same period of the day, weighted by the standard deviation of this specific heifer. The index values for activity change ranged from 0 to 100 (0 = lowest, 100 = highest). The period where the heifer’s activity change index exceeded 35 was considered as an estrus event. The onset of estrus was defined as the first time an activity change index of 35 was exceeded. The end of estrus was defined by the first instance at which the index fell below 35 again. For each AI, the peak activity and duration of estrus were calculated. Within an estrus event, estrous intensity was represented by the peak of activity. Estrous intensity was categorized into low (35–89 index value) and high (90–100 index value) peak activity, according to previous studies [12]. Estrous duration was defined as the interval from the onset to the end of estrus. In addition, estrous behavior was classified into heifers with a short inter-estrus interval and heifers with no short inter-estrus interval. No short inter-estrus intervals were defined as heifers with one period of increased activity within an observational period of 7 d around estrus (i.e., exceeding a threshold of 35, reaching an individual peak activity, and falling below 35 afterwards for one time). Short inter-estrus intervals were defined as heifers with more than one alert within an observational period of 7 d around the estrus (i.e., more than one peak of activity as activity change had a drop of below 35 in between the observational period). Due to our SCR data report settings regarding the observational period, short inter-estrus intervals were ranged between a minimum of 4 h and a maximum of 7 d. In the case of short inter-estrus intervals, the number of activity peaks was recorded and the period with the higher peak activity was used to calculate the duration, estrous intensity, and timing of AI in relation to an estrus event.

Files from the SCR system were generated in XLSX format, including heifer ID, last AI date, AI number, age at AI, and record of raw activity, daily activity, and activity change within the past 7 d every 2 h. Using DataFlow II, these files were exported on a weekly basis for all heifers that were bred within the last 7 d on each farm. The software tool BovHEAT v1.2.0, written in the open-source Python programming language (Python Software Foundation, Wilmington, DE, USA), was used to process all XLSX files into a single result report XLSX file [13].

### 2.3. Reproductive Management

The exclusion criteria after enrollment in the study were hormonal treatments before or at the time of AI (*n* = 436), such as PGF (*n* = 78) or GnRH (*n* = 358), the use of fresh semen (*n* = 614), embryo transfer (*n* = 20), more than one AI within <48 h (*n* = 44), AI before an activity alert, or AI with incomplete activity data (*n* = 151), and culling before a pregnancy diagnosis (*n* = 13). Most of the Holstein heifers were inseminated based on the alert of the AAM system. Heifers were considered eligible for breeding based on age (farms 1 and 4: minimum 13.5 months; farm 6: 14.5 months) and weight (all farms > 400 kg). A list of heifers eligible for breeding was generated by the AAM system, whereas all farms had their threshold for an activity alert set at an index of 35. In addition, the responsible AI technician verified heifers with an activity alert to be in estrus via the transrectal palpation of a highly contractile uterus and/or visualization of clear, stringy vaginal discharge. On farms 1, 3, 4, and 5, lists of heifers eligible for breeding were printed at approximately 7 a.m., and heifers were inseminated once daily (farm 1: at 11 a.m., farm 3: at 8 a.m., farm 4: at 2 p.m., farm 5: at 9 a.m.). On farms 2 and 6, lists of heifers eligible for breeding were printed twice daily (3 a.m. and 1 p.m.). Heifers in these herds were inseminated twice daily (in the early morning or in the afternoon). Heifers were inseminated with either conventional or sexed semen based on farm-individual strategies, whereas sexed semen was predominantly allocated to heifers receiving AI ≤ 3. Heifers from farm 1 remained in the study until they were considered pregnant, when they had no activity alert until 42 ± 3 d after AI. Since heifers from farm 1 were not routinely examined for pregnancy, calving dates were analyzed retrospectively to confirm their pregnancy status. As a reference, a combination of the mean (±2 SD) heifer’s gestation length of 278 ± 10 d [14,15] and an average interovulatory interval of 21 d [6] was used. Heifers were confirmed as pregnant if the interval between the day of AI and the day of calving was within the range of 278 ± 10 d and if they have had no prior or subsequent AI possibly related to this event. Heifers were confirmed as not pregnant if the interval between the day of AI and the day of calving that was larger than 278 ± 10 d. On farms 2 to 6, heifers remained in the study until a confirmed pregnancy diagnosis (Table 1), which was performed on a weekly basis via transrectal palpation 38 ± 3 d after AI (farm 3) or by transrectal ultrasound beginning 28 ± 3 d after AI (farms 2, 4–6). A verified pregnancy diagnosis was performed by trained personnel via transrectal palpation and included the presence of uterine fluid, asymmetry, and a positive fetal membrane slip. In the case of a transrectal ultrasound, a positive pregnancy diagnosis was based on the visualization of an embryo with a heartbeat. Non-pregnancy was based on the absence of pregnancy at the day of examination or a re-insemination to an estrus event before pregnancy diagnosis [16]. Heifers diagnosed as not pregnant were reassigned to AI after spontaneous estrus for a second AI or were moved to the bull pen for a third or greater AI (farm 5).

Sires were not randomly distributed across farms, and heifers were bred according to the breeding-matching program of the collaborating farms. Semen for this study was produced by genomically selected Holstein Friesian bulls from two local AI centers (RinderAllianz GmbH, Woldegk, Germany and Rinderproduktion Berlin-Brandenburg GmbH, Groß Kreutz, Germany). Ejaculates were obtained using an artificial vagina per standard operating procedure twice a week. Only ejaculates meeting the required quality standards (i.e., >70% progressive motility; >1.5 mL volume; >500 × 10^6^ spermatozoa) were used for the semen production and AI performed within this study. The inseminating doses were 20 × 10^6^ sperm/straw and 2 × 10^6^ sperm/straw for conventional and sexed semen, respectively. Semen was stored in 0.25-mL straws within a canister filled with liquid nitrogen at −196 °C and allocated to the farms as required. For thawing, straws were placed in a water bath at 37 °C for 15 s.

### 2.4. Data Collection and Statistical Analyses

Heifer ID, date of birth, AI date and time, number of AIs, AI code (i.e., AI according to AAM alert or hormone treatment), and type of semen (sexed or conventional) used for AI were obtained through the on-farm computer software (herdeW v5.8 or herdeplus; dsp agrosoft GmbH, Ketzin/Havel, Germany or DairyComp 305; DC305, Valley Agriculture Software, Tulare, CA, USA) or was documented on a list that was obtained on a monthly basis. All data were transferred to Excel (Microsoft Corporation, Redmond, WA, USA).

Through a post hoc analysis, we determined that the sample size necessary to detect (α = 0.05, β = 0.80) a 10.0 percentage unit difference (e.g., 45% vs. 55%) in P/AI among the different intervals within the onset, peak, or end of estrus to AI was 309 heifers per group. Furthermore, the sample size necessary to detect (α = 0.05, β = 0.80) a 5.0 percentage unit difference (e.g., 45% vs. 50%) in P/AI between type of semen (i.e., conventional vs. sexed) within a time interval was 1.233 heifers per group. All statistical analyses were performed using SPSS for Windows (version 28.0, SPSS Inc., IBM, Ehningen, Germany). Because of collinearity among the intervals (onset, peak, and end of estrus to AI), each of these estrus characteristics was tested separately to determine the optimum timing of AI. Therefore, 3 different logistic regression models, using the GENLINMIXED procedure of SPSS, were built. Farm was considered a random effect. Heifer was the experimental unit. The number of AIs was considered as repeated measure, as some heifers had more than one AI within the observational period. Model building was conducted as recommended by Dohoo et al. [17], where each variable was first analyzed separately in an univariable model. Only variables resulting in univariable models with *p* ≤ 0.10 were included in the final mixed model. The selection of the model that best fit the data was performed by using a backward stepwise elimination procedure that removed all variables with *p* > 0.10 from the model. The linear and quadratic associations between characteristics of an estrus event and P/AI were evaluated. The estimates of the analysis of maximum likelihood estimates from the logistic regression were used to generate probability curves. To better understand the associations among characteristics of an estrus event (i.e., onset, peak, and end) and timing of AI with P/AI, plausible intervals from the onset of estrus to AI (0 to 8 h, 9 to 16 h, 17 to 24 h, 25 to 32 h, and >32 h), peak of activity to AI (−8 to 0 h, 1 to 8 h, 9 to 16 h, 17 to 24 h, and >24 h) and the end of estrus to AI (−16 to −8 h, −7 to 0 h, 1 to 8 h, 9 to 16 h, and >16 h) were built. The initial model included the following explanatory variables as fixed effects: AI number (first AI vs. second AI vs. ≥third AI), month of AI (January to December), type of semen (conventional vs. sexed semen), estrous intensity (low vs. high), estrous duration (continuous), estrous behavior (no short inter-estrus interval vs. short inter-estrus interval), and the interval to AI (intervals from onset of estrus to AI vs. peak of activity to AI vs. end of estrus to AI).

We tested all biologically plausible 2-way interactions. Because there were no interactions between type of semen and peak activity (*p* = 0.44), type of semen and month of AI (*p* = 0.21), type of semen and AI number (*p* = 0.43), or peak activity and AI number (*p* = 0.44) with P/AI, these interactions were not included in the final statistical model. Regardless of the significance level, the type of semen, interval (onset, peak, or end of estrus) to AI, and interaction between the interval (onset, peak, or end of estrus) to AI and type of semen were forced to remain in the final model. The 3 final models for the association between P/AI and each interval (onset, peak, or end of estrus) to AI therefore contained the following fixed effects: AI number, type of semen, interval (onset, peak, or end) to AI, and the interaction between the type of semen and the respective interval to AI. A Bonferroni adjustment was used to account for multiple comparisons.

Variables were declared to be significant when *p* < 0.05. A statistical tendency was declared when *p* ≥ 0.05 and *p* ≤ 0.10.

## 3. Results

Overall, 3546 heifers were enrolled in this study, as well as activity information from 5543 inseminations. After the exclusion of 1384 AIs (25.0%), a total of 4159 inseminations representing 2858 heifers were included in the final statistical analyses. Inseminations were excluded due to hormonal treatments before AI (*n* = 436), AI before an activity alert or with incomplete activity data (*n* = 151), the use of fresh semen (*n* = 614), embryo transfer (*n* = 20), more than one AI within <48 h (*n* = 44), and culling before a pregnancy diagnosis (*n* = 13). In addition, AIs from farm 1 were excluded (*n* = 106) if they had no valid information on the calving event afterwards, such as AIs from heifers that died before calving (*n* = 62), heifers whose calving dates could not be retraced in the herd’s management software (*n* = 27), inseminations performed although pregnancy was already established after a previous AI (*n* = 9), and in the case of two consecutive inseminations for which the success of the AI was not clearly attributable to one of the two AI (*n* = 8).

The mean (±SD) duration of estrous activity was 16.1 ± 5.2 h. The mean interval from the onset of estrus to AI was 16.9 ± 7.6 h, from the peak of activity to AI, 10.9 ± 7.8 h, and from the end of estrus to AI, 0.8 ± 8.7 h. The mean index value of peak activity was 91.4 ± 12.1, whereas 33.0% of heifers had low and 67.0% had high estrous intensity.

### 3.1. Interval from Onset of Estrus to AI

**Figure 1:**
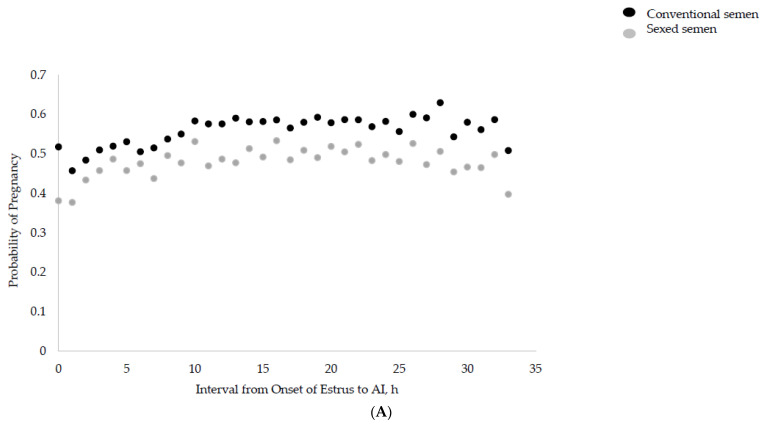

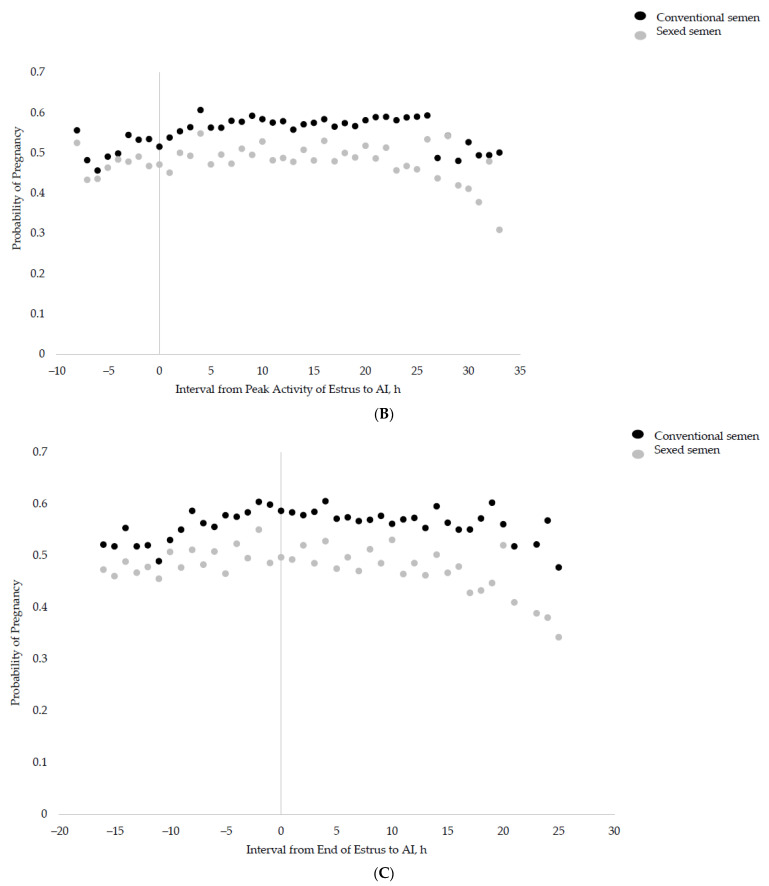
Probability of pregnancy for heifers (*n* = 2858) inseminated with either conventional semen (black dots) or sexed semen (grey dots) considering the interval from (1) onset of estrus to AI (Panel (**A**)), (2) peak activity of estrus to AI (Panel (**B**)), and (3) end of estrus to AI (Panel (**C**)) using a neck-mounted accelerometer system for estrus detection (Heatime; SCR Engineers Ltd., Netanya, Israel). The onset of estrus was defined as the first time an activity change index of 35 was exceeded. The intensity of an estrus was represented by the peak of the activity change index value during an estrus event. The end of estrus was defined by the first instance in which the index fell below 35 again. Linear and quadratic associations between characteristics of an estrus event (i.e., onset, peak, and end of estrus) and P/AI were evaluated by building 3 different logistic regression models using the GENLINMIXED procedure of SPSS. Farm was considered a random effect. Heifer was the experimental unit. The number of AIs was considered as a repeated measure as some heifers had more than one AI within the observational period. The estimates of the analysis of maximum likelihood estimates from the logistic regression were used to generate probability curves (Panel (**A**): linear, *p* = 0.03; quadratic, *p* = 0.03; Panel (**B**): linear, *p* = 0.21; quadratic, *p* = 0.13; Panel (**C**): linear, *p* = 0.99; quadratic, *p* = 0.02).

**Figure 2:**
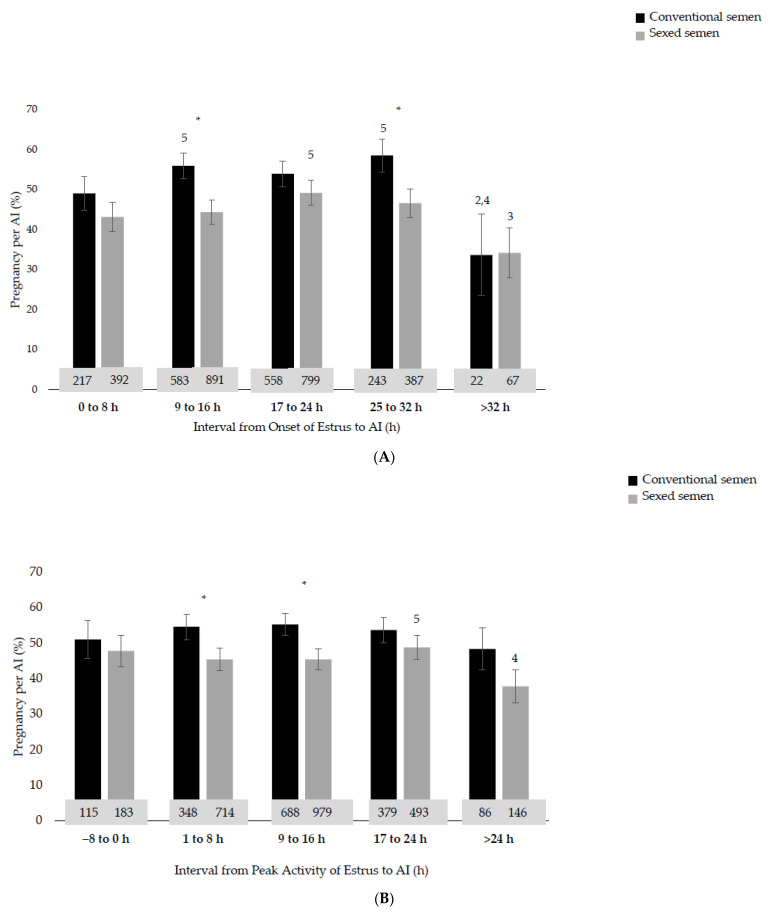

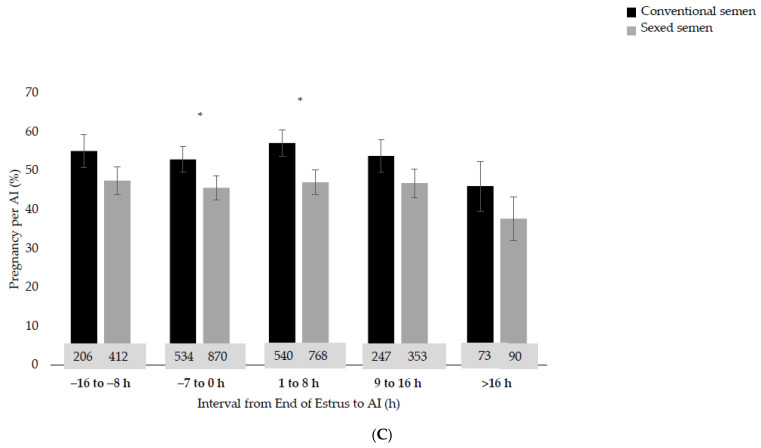
Pregnancy per artificial insemination (P/AI) (±SEM) for heifers (*n* = 2858) inseminated with either conventional semen (black bars) or sexed semen (grey bars) considering the interval from (1) onset of estrus to AI (Panel (**A**)), (2) peak activity of estrus to AI (Panel (**B**)), and (3) end of estrus to AI (Panel (**C**)) using a neck-mounted accelerometer system for estrus detection (Heatime; SCR Engineers Ltd., Netanya, Israel). The onset of estrus was defined as the first time an activity change index of 35 was exceeded. The intensity of an estrus was represented by the peak of the activity change index value during an estrus event. The end of estrus was defined by the first instance at which the index fell below 35 again. A Bonferroni adjustment was used to account for multiple comparisons. An asterisk indicates a significant difference (*p* < 0.05) between type of semen (i.e., conventional vs. sexed semen) within a time interval. Bars with different numbers indicate a significant difference (*p* < 0.05) among time intervals for either conventional or sexed semen. Therefore, time intervals were classified into numbers (1 to 5), with number 1 beginning at the first time interval in ascending order. The number within each bar is the number of heifers inseminated with this type of semen within that specific time interval.

The interval from the onset of estrus to AI was associated with the P/AI (linear, *p* = 0.03; Figure 1A). In addition, we detected a quadratic association between the interval from the onset of estrus to AI and the P/AI (*p* = 0.02), as heifers inseminated from 9 to 32 h after the onset of estrus had the greatest P/AI. Heifers inseminated within 8 h after the onset of estrus had similar P/AIs (*p* = 0.12) compared with heifers inseminated within 9 to 16 h after the onset of estrus, but a decreased P/AI compared with heifers inseminated within 17 to 32 h after the onset of estrus (*p* < 0.05). Heifers receiving AI 32 h or later after the onset of estrus had lower P/AIs (0 to 8 h: 46.0%; 9 to 16 h: 50.1%; 17 to 24 h: 51.5%; 25 to 32 h: 52.5%; >32 h: 33.8%). There was no interaction (*p* = 0.34) between the interval from the onset of estrus to AI and the type of semen (Figure 2A).

### 3.2. Interval from Peak of Activity to AI

The interval from the peak of activity to AI was not associated with the P/AI (linear, *p* = 0.21; Figure 1B). In addition, we found no association between the interval from the peak of activity to AI and the P/AI (−8 to 0 h: 49.3%; 1 to 8 h: 50.0%; 9 to 16 h: 50.3%; 17 to 24 h: 51.2%; >24 h: 43.0%; *p* = 0.32). We observed no interaction between the interval from the peak of activity to AI or the type of semen and the pregnancy outcome (*p* = 0.69; Figure 2B).

### 3.3. Interval from End of Estrus to AI

The interval from the end of estrus to AI was not associated with the P/AI (linear, *p* = 0.99; Figure 1C). In addition, we found no association between the interval from the end of estrus to AI and the P/AI (−16 to −8 h: 51.3%; −7 to 0 h: 49.2%; 1 to 8 h: 52.1%; 9 to 16 h: 50.3%; >16 h: 41.7%; *p* = 0.14). We observed no interaction between the interval from the end of estrus to AI or the type of semen and the pregnancy outcome (*p* = 0.96; Figure 2C).

### 3.4. Factors Associated with P/AI

Overall, the P/AI was 46.7%. The heifers inseminated with conventional semen (50.1%; 926/1623) had greater P/AIs compared with the heifers inseminated with sexed semen (43.3%; 1234/2536; *p* = 0.03). The mean age at first AI was 452.9 ± 43.6 d and the mean age at AI was 467.0 ± 51.3 d. A percentage of 61.9% of the AIs were first inseminations, whereas 26.8% were second and 11.3% were third or greater AIs. The number of AIs was associated with the P/AI (*p* < 0.01), as heifers receiving their third or more AI (40.4%; *p* < 0.01) had a lower P/AI compared with heifers receiving their first (51.0%) or second AI (48.7%). The distribution of the type of semen among the participating farms depending on the number of AIs is depicted in Table 2. The month of an AI was not associated (*p* = 0.12) with the P/AI. The pregnancy per AI was not associated with estrous intensity (low intensity: 50.5% vs. high intensity: 52.0%; *p* = 0.37). A small percentage of heifers (3.1%) had short inter-estrus intervals. The heifers with short inter-estrus intervals (49.3%) had similar P/AIs compared with heifers with no short inter-estrus intervals (51.5%; *p* = 0.70).

## 4. Discussion

The objective of this study was to determine the association between the timing of an AI and the P/AI in Holstein heifers inseminated with either sexed or conventional semen using an AAM system. Furthermore, we wanted to assess the association between the intensity of estrous and reproductive performance. We expected that delaying AI after the onset of estrus would improve the P/AI in heifers inseminated with sexed semen compared with conventional semen.

The mean (±SD) duration of estrous activity was 16.1 ± 5.2 h, which is in agreement with other studies using AAM systems in Holstein heifers [3,18]. It is widely recognized that early insemination (i.e., close to the onset of estrus), thus further away from presumptive ovulation, results in a reduced fertilization rate but good embryo quality [2]. Late insemination, on the other hand, is assumed to have exactly the opposite effect (i.e., greater fertilization rate but reduced embryo quality) [2]. The timing of an AI in relation to the presumptive time of ovulation therefore is essential for adequate pregnancy outcomes. Three studies investigating AAM systems in heifers were able to achieve similar results with respect to the interval from the onset of estrus to ovulation ranging from 25 to 29 h [3,4,19]. We observed a quadratic association between the interval from the onset of estrus to an AI and the P/AI, as heifers inseminated from 9 to 32 h after the onset of estrus had the greatest P/AI. Therefore, automated activity monitors represent an opportunity to investigate the optimum timing of AI relative to the specific characteristics of an estrus event (i.e., the onset, peak, and end of estrus) in heifers, even though there has not been as much research in this area.

Overall, in our study, the heifers inseminated with conventional semen had approximately seven percentage units greater P/AIs compared with the heifers inseminated with sexed semen. Sexed semen fertility was commonly reported to be within 80% of the conventional semen fertility [20] but ranging from ≤55% to 90% in studies where heifers were inseminated following estrus detection [10,21,22]. The frequently discussed causes for a reduction in fertility are individual bull differences with regard to the sorting process, the lower sperm concentration for straws containing sexed semen, and farm-individual reproductive management strategies [23,24,25]. In general, however, studies from different years should be compared with caution, as there has been a refinement of the sex-sorting technology in the three decades since its inception. A recent meta-analysis [26], including 45 field studies from 1999 to 2021, evaluated the reproductive success of bovine sexed semen and provided evidence that the refinement of sex-sorting technology resulted in increased P/AIs. The lifespan of sexed semen in the female reproductive tract is still a matter of debate, but the sorting process was described to induce a partial capacitation in sperm, resulting in a shortened time span for them to become functional for fertilization [24]. Therefore, the optimum timing of AI relative to the onset of estrus might differ between sexed and conventional semen. However, we did not observe an association between the type of semen and the timing of an AI. Several studies suggested performing AI with sexed semen closer to the time of ovulation [3,9,27]. Inseminating Jersey heifers 6.5 h versus 12.5 h before presumptive ovulation was demonstrated to increase the P/AI when using sexed but not conventional semen [8]. Accordingly, for conventional semen, each hour increase in the interval from the onset or peak of estrus to AI reduced the predicted probability of pregnancy in heifers detected in estrus by an AAM system [4]. Conversely, for sexed semen, they observed no association between the intervals from the onset or peak of estrus to AI and the predicted probability of pregnancy. This was partly confirmed in another study where heifers were detected to be in estrus by an activity monitor after their enrollment in a 5 d Cosynch protocol [10]. The pregnancy per AI for heifers inseminated with sexed semen was not improved when the AI was delayed by approximately 12 h. However, the authors assumed a limited ability to properly test whether delaying AI would improve the P/AI, as they chose too large intervals from the onset of estrus to AI.

Approximately two thirds of the heifers (67.0%) expressed high estrous intensity, whereas one third (33.0%) had low estrous intensity. This is interesting as heifers are not or are less affected by many factors described as being associated with estrous expression in dairy cows, such as lactation stage, udder health, metabolic diseases, or disorders of the locomotion system [11,28]. Recent studies found high peak activity to be a parameter suitable for the selection of dairy cows to optimize the P/AI [11,12]. Contrary to our expectations, however, estrous intensity was not associated with the P/AI in heifers from our study. Moreover, two further studies were unable to establish an association between the estrous intensity measured by an AAM system and the P/AI in heifers [4,29]. Others, however, indicated that the expression of estrus at the end of a TAI protocol would be favorable for fertility of heifers [27,30]. The underlying physiological mechanisms regarding this association should be part of more detailed investigations. Increased progesterone concentrations around the time of estrus were reported to block the effects of estradiol, which is responsible for estrous behavior as well as for fertilization and early embryonic development [31,32,33]. Nevertheless, there is evidence that estradiol concentrations were not correlated with estrous intensity but rather, when exceeding a certain threshold concentration of estradiol, were decisive to inducing estrous behavior [18,34,35]. However, studies on the association between estrous intensity and the physiological mechanisms in heifers are rare.

In the current study, the heifers’ mean age at the time of AI was approximately 15.5 months, and heifers receiving their third or greater AI had lower P/AIs compared with heifers receiving their first or second AI. After evaluating more than 537,900 inseminations in 2668 herds from 41 United States, Kuhn et al. [36] reported the maximum P/AI in heifers at an intermediate age of 15 to 16 months, with lower fertility for heifers inseminated at older ages and an increasing AI number. There is clear evidence that farms using estrus detection aids manage to reduce the age of the heifers at the time of their first AI, the interval between their first and second AI, and the age at their first calving [37,38]. In combination with heifer’s age, body weight is an important indicator for farms to initiate the first AI, as it was also shown to be linked to subsequent fertility [39]. In this context, heifer nutrition during early and mid-pregnancy is of major importance regarding the establishment and maintenance of pregnancy, as diet was found to be associated with oocyte quality, embryo development, and fetal growth [40,41,42]. However, the assessment of a heifer’s body weight at AI and the evaluation of farm-individual diets was not feasible in regard to our study design, which included quite a large sample size from different farms. Furthermore, the season of the AIs was detected as one of the most important factors for the variation in the P/AI in heifers [36,43]. The month of the AIs, however, was not associated with the P/AI in the heifers from our study. Similar observations were made in previous studies, where heifer fertility is nearly as good in the warmer as in the cooler months [3,36]. In fact, Sartori et al. [44] noticed a lower increase in the body temperature of heifers than in that of cows when the ambient temperature was increasing.

Due to the nature of a retrospective, observational study, one limitation was that there was no option of randomizing the type of semen and the timing of AI after the activity alert. The external validity was improved by enrolling heifers from six farms, thereby broadening the understanding about the use of AAM-based estrus detection in heifers. Another limitation of our study was that the heifers were not confirmed as being in estrus through a transrectal ultrasonography of the ovaries or by measuring serum progesterone concentrations after the activity alert. Moreover, it is relevant to consider the potential for a misclassification of the pregnancy outcome of heifers from farm 1. Due to their standard procedure (i.e., heifers were considered pregnant when they had no further activity alert until 42 ± 3 d after AI), the subsequent calving date was the main determinant of AI success. This might have caused a certain bias, since heifers with presumed pregnancy loss were excluded from the final analysis compared to heifers that might have experienced pregnancy loss from the other farms. There are only a few studies on pregnancy loss in heifers, but they show evidence that the percentage of pregnancy loss is about 8% in heifers [45,46]. Furthermore, we tried to minimize misinterpretation by using a combination of studies evaluating large datasets on the gestation length in heifers as a reference and by excluding AI from the final analysis, whose success could not be clearly traced on the basis of calving data. In this context, it is interesting to note that, through examining AAM systems in heifers, a positive predictive value of approximately 84% and a sensitivity of 91% were revealed based on the analysis of ovarian dynamics (i.e., ovulation) [4,18]. These investigations support the fact that AAM systems represent a useful and, to a large extent, reliable tool to assist in heifers’ reproductive management.

## 5. Conclusions

Using an AAM system, we found no evidence for a strict timing of AI in heifers, as consistently great P/AIs were seen for a wide time span of 9 to 32 h after the onset of estrus, irrespective of the type of semen used (conventional vs. sexed). Furthermore, we found no association between the interval from the peak of activity to an AI and the P/AI or between the interval between the end of the estrus to an AI and the P/AI. About two thirds of heifers expressed high estrous intensity, which, however, was not associated with the pregnancy outcomes. Further research is warranted to investigate the latter association in more detail and to elucidate the corresponding physiological mechanisms in heifers. In addition, more controlled studies should continue to identify risk factors in order to improve reproductive performance in heifers inseminated with sexed semen.

## Figures and Tables

**Table 1:** Descriptive information of the reproductive herd status from enrolled farms.

Parameter	Farm					
	1	2	3	4	5	6
Average farm size	-	716	427	581	594	515
Average 305 d milk yield (kg)	-	9677	9461	9105	9500	9975
Average number of replacement heifers	868	729	395	453	487	495
Age at first service (months, ±SD)	15.0 ± 1.3	14.1 ± 1.7	15.5 ± 1.4	14.2 ± 0.9	14.0 ± 0.7	16.1 ± 0.7
Pregnancy per AI at 1st service (%)	63	50	57	46	54	49
Overall pregnancy per AI (%)	59	40	62	49	49	53
Heat detection rate (%)	69	78	41	75	62	77
Day of pregnancy diagnosis after AI ^2^	42 ± 3	28 ± 3	38 ± 3	32 ± 3	30 ± 3	30 ± 3
Method of pregnancy diagnosis	Alert of the AAM ^1^ system	Transrectal ultrasound	Transrectal palpation	Transrectal ultrasound	Transrectal ultrasound	Transrectal ultrasound
Age at first calving (months, ±SD)	26.5 ± 1.7	25.0 ± 1.7	25.5 ± 1.6	23.6 ± 1.3	23.0 ± 0.6	26.0 ± 0.9

^1^ All heifers were equipped with neck-mounted automated activity monitor (AAM) systems for detection of estrus (Heatime; SCR Engineers Ltd., Netanya, Israel). ^2^ On each farm, pregnancy diagnosis was performed on a weekly basis.

**Table 2:** Distribution of type of semen [% (No./No.)] among participating farms depending on the number of AIs.

Farm	1st AI		2nd AI		≥3rd AI	
	Conventional Semen	Sexed Semen	Conventional Semen	Sexed Semen	Conventional Semen	Sexed Semen
1	10.7 (70/657)	89.3 (587/657)	13.0 (41/315)	87.0 (274/315)	78.3 (76/97)	21.7 (21/97)
2	60.3 (414/686)	39.7 (272/686)	79.6 (219/275)	20.4 (56/275)	-	-
3	19.5 (69/354)	80.5 (285/354)	35.5 (54/152)	64.5 (98/152)	85.6 (83/97)	14.4 (14/97)
4	22.9 (128/559)	77.1 (431/559)	52.7 (129/245)	47.3 (116/245)	77.3 (167/216)	22.7 (49/216)
5	12.9 (21/163)	87.1 (142/163)	4.7 (2/43)	95.3 (41/43)	27.3 (3/11)	72.7 (8/11)
6	34.0 (52/153)	66.0 (101/153)	59.3 (51/86)	40.7 (35/86)	88.0 (44/50)	12.0 (6/50)
Total	28.9 (759/2623)	71.1 (1864/2623)	43.3 (500/1154)	56.7 (654/1154)	78.9 (385/488)	21.1 (103/488)

## Data Availability

The data are contained within the article. The raw data are available on request from the corresponding author.
